# Does the Use of Denosumab in Combination with bDMARDs or tsDMARDs Increase the Risk of Infection in Patients with Osteoporosis and Inflammatory Rheumatic Diseases?

**DOI:** 10.3390/jcm14176090

**Published:** 2025-08-28

**Authors:** Salim Mısırcı, Ali Ekin, Burcu Yağız, Belkıs Nihan Coşkun, Mustafa Çağatay Büyükuysal, Ediz Dalkılıç, Yavuz Pehlivan

**Affiliations:** 1 Department of Internal Medicine, Division of Rheumatology, Faculty of Medicine, Bursa Uludag University, Bursa 16059, Turkey; aliekin49@hotmail.com (A.E.); burcuyilmaz_84@hotmail.com (B.Y.); belkisnihanseniz@hotmail.com (B.N.C.); edizinci@hotmail.com (E.D.); pehlivany@uludag.edu.tr (Y.P.); 2 Department of Biostatistics, Faculty of Medicine, Zonguldak Bülent Ecevit University, Zonguldak 67630, Turkey; cbuyukuysal@gmail.com

**Keywords:** bDMARD, denosumab, infection, osteoporosis, tsDMARD

## Abstract

**Background/Objectives:** The combination of denosumab treatment with biological disease-modifying antirheumatic drugs (bDMARDs) or targeted synthetic disease-modifying antirheumatic drugs (tsDMARDs) in patients with inflammatory rheumatic diseases (IRD) may raise safety concerns for clinicians, particularly regarding infections. In this study, we investigated whether the risk of infection increases in patients who receive bDMARDs or tsDMARDs for IRD and are simultaneously treated with denosumab for osteoporosis (OP). **Methods:** As a control group, we evaluated patients receiving bDMARDs or tsDMARDs concomitantly with zoledronic acid (ZA), which is not clearly associated with infections. A total of 88 patients—including 30 patients receiving bDMARDs or tsDMARDs with ZA and 58 patients receiving bDMARDs or tsDMARDs with denosumab—met the criteria and were included in this study. The groups were compared in terms of the ratio/risk of serious infections requiring hospitalisation and infections requiring outpatient treatment after applying the inverse probability of treatment weighting (IPTW) to the control for confounding factors. The Cox proportional hazards regression model was used to compare the risks of infection, taking confounding factors into account. **Results:** The mean age of patients in the ZA group was 59.07 years (±SD: 13.65), while that of patients in the denosumab group was 69.93 years (±SD: 11.72). Comorbidities occurred more frequently in the denosumab group (*n* = 44, 75.86%) than in the ZA group (*n* = 14, 46.66%). The median duration of medication use in the denosumab group was 24 months (minimum: 6 months; maximum: 72 months). The median duration of medication use in the ZA group was 24 months (minimum: 12 months; maximum: 60 months). When comparing the groups regarding the risk of infection, denosumab was not associated with an increased risk of either a serious infection requiring hospitalisation (Hazard Ratio (HR): 0.37; 95% Confidence Interval (CI): 0.14–0.94) or an infection requiring outpatient treatment (HR: 0.29; 95% CI: 0.12–0.71). **Conclusions:** In conclusion, the combination of denosumab treatment with bDMARD or tsDMARD treatments is safe for short-term use.

## 1. Introduction

Osteoporosis (OP) is a systemic, metabolic bone disease characterised by a reduced bone density and impaired microarchitecture. The prevalence of OP is around 19.7% and increases with age [1].

OP, one of the most common comorbidities in the course of inflammatory rheumatic disease (IRD), has a multifactorial aetiology. In addition to genetics, age and gender, many factors such as inflammation, immobilisation and medical treatments (especially glucocorticoids (GCs)) pose a risk for OP [2,3]. Assessing the frequency of OP that may occur during IRD, it is quite common and has been found to occur in 30–50% of patients with rheumatoid arthritis (RA), in 1.4–68% of patients with systemic lupus erythematosus (SLE) and 18.7–62% of patients with ankylosing spondylitis [4,5,6].

With the progression of OP, back pain, osteoporotic fractures and spinal deformities may develop. This condition leads to limited mobility, a low quality of life and may cause physical and psychological complications for patients. Hip fractures in particular are a significant cause of mortality in OP patients. As a result, OP is a major cause of morbidity and mortality for patients, while also imposing a high economic burden on healthcare services [7,8,9].

Zoledronic acid (ZA) and denosumab are anti-resorptive agents that have been used for many years to treat OP. ZA is a pyrophosphate analogue from the bisphosphonate group which inhibits osteoclastic activity by binding to hydroxyapatite crystals. A clear connection with infection is not known [10]. Denosumab is a human monoclonal antibody that inhibits bone resorption by binding to the nuclear factor-κB receptor activator (RANK) ligand (RANKL) and preventing interactions between RANKL and RANK, the receptor on the surface of osteoclasts and their precursors [11,12,13].

During the remodelling of normal bone, damaged tissue is broken down by osteoclasts and new bone is formed by osteoblasts. This process is controlled by osteocytes, which regulate bone formation through the expression of the Wnt inhibitors sclerostin and Dickkopf-1 and regulate bone resorption through the expression of RANKL and osteoprotegerin (OPG). OPG acts as a trap receptor that binds to RANKL and prevents its interaction with RANK [11].

When evaluating the infection rates associated with denosumab, the FREEDOM study—a placebo-controlled trial involving postmenopausal women aged 60–90 years with OP—found that the overall infection rate in the denosumab group was 52.9% and the rate of serious infections was 4.1% [14]. In a study in which 3185 patients were treated with denosumab, most of whom were women (93.3%), the rate of serious infections was 1.4% [15]. In another randomised controlled study in women over 55 years of age, in which treatment with risedronate and denosumab was compared, the rate of serious infections in both groups was 1.2% [16].

RANKL is also expressed by immune system cells, including synovial fibroblasts and macrophages, activated T and B lymphocytes and dendritic cells outside the bone. Therefore, the inhibition of RANKL and its effects on cells of the immune system theoretically suggest that the risk of infection could increase. However, it is assumed that the RANKL–RANK signalling pathway plays a secondary role in influencing the intensity of the inflammatory response rather than a primary role in the immune system [17,18,19].

In the course of an IRD, the risk of infection is increased compared with the general population due to age, comorbidities, disease-related immunological disorders and the medication used [20]. In particular, the combined use of biologic disease-modifying antirheumatic drugs (bDMARDs) and/or targeted synthetic disease-modifying antirheumatic drugs (tsDMARDs) in patients with resistant IRDs should be considered. However, this raises safety concerns, particularly regarding infections [21,22]. Denosumab, a monoclonal antibody that inhibits RANKL, may be favoured due to OP, which is a common comorbidity in the course of IRDs. The combination of denosumab with bDMARDs or tsDMARDs could theoretically increase infection rates. However, there are only a few studies on this topic in the literature [10,23,24].

In this study, we investigated whether the risk of infection increased in patients who received bDMARDs or tsDMARDs for IRD and were simultaneously treated with denosumab for OP. As a control group, we evaluated patients receiving bDMARD or tsDMARD treatment and concomitant ZA treatment, which is not clearly associated with infection.

## 2. Materials and Methods

### 2.1. Study Design and Patients

Our study included patients aged 18 years and older who were followed up between June 2014 and June 2024 at the Department of Rheumatology, Faculty of Medicine, Uludağ University, Bursa, and who had been treated with either denosumab or ZA in combination with bDMARD or tsDMARD. The patients were retrospectively screened from hospital records (Ethics Committee Approval: 30 October 2024; protocol code: 2024-17/6).

Patients with a follow-up period of at least 6 months after denosumab (60 mg) and a follow-up period of at least 12 months after ZA (5 mg) were included. The denosumab dose approved for OP treatment and reimbursable under our country’s healthcare circular is 60 mg/subcutaneous/6 months, while the ZA dose is 5 mg/intravenous/year. The patients were evaluated using dual-energy X-ray absorptiometry (DEXA) before planning the OP treatment to ensure that they fulfilled the reimbursement conditions of our country’s healthcare system. The decision for OP treatment in our rheumatology clinic is based on current DEXA results (T-scores of the femoral neck, total hip, or spine), the presence of previous or current major osteoporotic fractures and the fracture risk. The T-scores for OP treatment in patients without a history of major osteoporotic fractures were ≤−3 in patients aged <65 years (postmenopausal women and men aged 50 years and older) and ≤−2.5 in patients aged >65 years. In patients with a history of major osteoporotic fractures, OP treatment was performed in patients with a T-score ≤−1. Patients had to have taken at least one bDMARD or tsDMARD for at least 3 months in combination with denosumab or ZA. The bDMARD and tsDMARD doses were as follows: Infliximab (5 mg/kg/6 weeks), Abatacept (125 mg/s.c/week), Adalimumab (40 mg/s.c/2 weeks), Certolizumab (200 mg/s.c/2 weeks), Etanercept (50 mg/s.c/week), Golimumab (50 mg/s.c/month), Rituximab (1000 mg/i.v./every 6 months on days 0–15), Tocilizumab (162 mg/s.c/4 weeks), Secukinumab (150 mg/s.c/month) and Tofacitinib (10 mg/oral/day or 11 mg/oral/day).

A total of 88 patients, including 30 receiving bDMARD–tsDMARD and ZA simultaneously and 58 receiving bDMARD–tsDMARD and denosumab simultaneously, met the criteria and were included in this study.

The patients were divided into two groups and compared: those who used denosumab in combination with bDMARD–tsDMARD treatments and those who used ZA in combination with bDMARD–tsDMARD treatments.

### 2.2. Clinical and Laboratory Variables

Demographic characteristics, comorbidities, the IRD diagnosis and duration (months) of denosumab and ZA use were recorded. The bDMARD–tsDMARD treatments and their duration, the use of conventional disease-modifying antirheumatic drugs (csDMARDs) and the use of GCs and their dosage (prednisolone ≤ 5 mg equivalent dose or prednisolone > 5 mg equivalent dose) were recorded.

The presence and causes of infections requiring hospitalisation (with intravenous antibiotic treatment) or outpatient treatment (with appropriate oral antibiotic treatment) were recorded.

### 2.3. Statistical Analysis

The compatibility of the continuous variables with the normal distribution was tested using the Shapiro–Wilk and Kolmogorov–Smirnov tests. Continuous variables that did not show normal distribution were expressed as the median (minimum; maximum) and categorical variables were expressed as n (%). When comparing two groups, the Mann–Whitney U test was used for continuous variables that did not have a normal distribution. The Pearson and Fisher Chi-square tests were used to compare categorical variables. An inverse probability of treatment weighting (IPTW) was applied to avoid confounding factors such as age, chronic kidney disease (CKD), hypertension (HT), coronary artery disease (CAD) and GC use. The Cox proportional hazards regression model was used to compare the risks of infection, taking into account confounding factors. The SPSS (version 28.0; IBM Corporation, Armonk, NY, USA) package program was used for statistical data calculations. *p* < 0.05 was accepted as the statistical significance level.

## 3. Results

### 3.1. Clinical and Demographic Baseline Data of the Denosumab Group and the ZA Group

The mean age of patients in the ZA group (*n =* 30) was 59.07 (±SD: 13.65) and the mean age of patients in the denosumab group (*n* = 58) was 69.93 (±SD: 11.72). The patients in the denosumab group were thus older (*p* < 0.001). When comparing gender, female patients predominated in both groups and no significant difference was found between them (*p* = 0.138).

RA was the most common diagnosis in both groups and occurred more frequently in the denosumab group (*p* = 0.017). The median duration of drug use in the denosumab group was 24 months (minimum: 6 months; maximum: 72 months). The median duration of drug use in the ZA group was 24 months (minimum: 12 months; maximum: 60 months).

In the assessment of comorbidities, HT was the most common in both groups (ZA: *n* = 12, 40%; denosumab: *n* = 35, 65.51%). However, more comorbidities were found in the denosumab group (*n* = 44, 75.86%) than in the ZA group (*n* = 14, 46.66%) (*p* = 0.006). In addition, no CAD, CKD, hyperlipidaemia or congestive heart failure were found in the ZA group.

When comparing bDMARDs and tsDMARDs taken for an existing IRD, no significant difference was found between the groups (*p* > 0.05). The most commonly used bDMARD in both groups was rituximab (RTX). There was no significant difference in csDMARDs used in combination with bDMARDs or tsDMARDs. However, the proportion of patients using low-dose GCs (prednisolone ≤5 mg equivalent dose) was higher in the ZA group (ZA: *n* = 22, 73.33%; denosumab: *n* = 28, 48.27%) (*p* = 0.024) (Table 1).

When IPTW was applied, taking into account confounding factors such as age, CKD and GC use, the use of (RTX), tofacitinib and hydroxychloroquine was higher in the ZA group, while the use of abatacept, etanercept and leflunomide was higher in the denosumab group (*p* < 0.05) (Table 2).

### 3.2. Comparison of Infections in Patients Receiving Denosumab and ZA

When comparing the ZA and denosumab groups, considering the rate of serious infections requiring hospitalisation and infections requiring outpatient treatment after IPTW application, infection rates were statistically significantly higher in the ZA group (*p* = 0.003 and *p* = 0.007, respectively). The rates of severe pneumonia requiring hospitalisation and urinary tract infections requiring outpatient treatment were also higher in the ZA group (*p* < 0.001 for both). The comparison of patients with regard to infections is shown in Table 3. When evaluating the risk of infection using the Cox proportional hazards regression model, taking into account confounding factors, denosumab treatment was not associated with the risk of severe infection requiring hospitalisation (Hazard Ratio (HR): 0.37; 95% Confidence Interval (CI): 0.14–0.94). It was also not associated with an increased risk of infection requiring outpatient treatment (HR: 0.29; 95% CI: 0.12–0.71). On the other hand, the use of a >5 mg prednisolone equivalent dose in the denosumab group significantly increased the risk of infection requiring outpatient treatment (HR: 8.6; 95% CI: 1.5–48.7).

## 4. Discussion

We compared IRD patients who received bDMARDs or tsDMARDs in combination with denosumab with IRD patients who received bDMARDs or tsDMARDs in combination with ZA. When the patients in the denosumab group were compared with the patients in the ZA group regarding the risk of outpatient infections and infections requiring hospitalisation, the use of denosumab did not increase the risk of infection. However, both the rates of serious infections requiring hospitalisation and the rates of infections requiring outpatient treatment were high in the ZA group. When analysing the causes of infection, the rates of severe pneumonia requiring hospitalisation and urinary tract infections requiring outpatient treatment were also higher in the ZA group.

The inhibition of RANK and RANKL—which are expressed by cells of the immune system such as T lymphocytes and B cells—by denosumab raises concerns about an increased risk of infection [11]. RANK is also expressed on dendritic cells, which play an important role in activating T cells [25]. Given the current pathophysiology, the infection rates in studies on denosumab treatment were also evaluated with regard to side effects [14,25,26,27,28]. GCs, csDMARDs, bDMARDs and tsDMARDs are used to treat patients with IRD, especially RA, and new agents continue to be investigated. The agents used in IRD treatment inhibit cytokines involved in the pathogenesis of IRD, block T cell costimulation, deplete B cells and inhibit the janus kinase pathway [29]. The combination of these agents raises concerns about the risk of infection, considering the inhibition of RANK and RANKL expressed in cells of the immune system by denosumab treatment and the inhibition of signalling pathways important and necessary for immune system function by the treatments used in IRD therapy.

In a placebo-controlled denosumab study (FREEDOM), there was no difference in the overall frequency of the side effects between the denosumab group and the placebo group, but cellulitis and eczema occurred more frequently in the denosumab group. When comparing the infections, the overall infection rate was 52.9% in the denosumab group and 54.4% in the placebo group. The rate of serious infections was 4.1% in the denosumab group and 3.4% in the placebo group, with no statistically significant difference between the groups (*p*-values: 0.17 and 0.14, respectively) [14].

In a cohort study investigating community-acquired pneumonia, patients treated with denosumab (*n =* 933) were compared with patients treated with alendronate (*n* = 4652). After a follow-up period of 5 years, the incidence of community-acquired pneumonia per 1000 person-years was 72.0 (95% CI, 60.1–85.7) in the denosumab group and 75.1 (95% CI, 69.4–81.2) in the alendronate group. In conclusion, treatment with denosumab did not lead to a significant increase in community-acquired pneumonia [26]. In our study, there was no increase in the pneumonia rate in the denosumab group compared with the control group. However, a national cohort study reported that the risk of infection increased in the initial phase of denosumab treatment, but decreased significantly after the second year of treatment [25].

In a study comparing patients over 50 years of age with ZA and denosumab, treatment with denosumab was not associated with an increased risk of serious infections (HR: 0.81; 95% CI: 0.55–1.21) [27]. The results of our study are consistent with the literature. A meta-analysis evaluating cohort studies of patients who received ZA and denosumab also found no significant difference in the number of adverse events and risk of serious infection [28].

If resistance to therapy develops in IRD patients, a combination of bDMARD and/or tsDMARD treatments may be considered, although safety concerns are paramount. The COMBIO study, which involved 143 patients, investigated the efficacy and safety of targeted combination therapies for immune-mediated inflammatory disease (IMID) [21]. The most common diagnoses were Crohn’s disease (63.6%), axial spondyloarthritis (37.7%) and ulcerative colitis (14%). Combinations with the anti-tumour necrosis factor (TNF) agents vedolizumab (30%) or ustekinumab (28.7%) were used most frequently. The incidence of serious infections was 4.51 per 100 person-years (95% CI 2.20–8.27). As a result, this rate did not increase, compared with monotherapy with bDMARDs, and that combination treatment may be safe in patients with IMID. However, a meta-analysis of 10 randomised controlled trials comparing combination therapies with monotherapies in patients with IMID reported that there was no clinical benefit in patients with rheumatological IMID and a higher incidence of side effects in RA [22].

Mirzaei et al. [23] investigated RA patients with postmenopausal OP. RA patients who received combined treatment with denosumab and bDMARD (*n* = 40) were compared with patients who received bDMARD alone (*n* = 44) regarding the risk of infection. Serious infections occurred in two patients in both groups, and there was no significant difference between the groups in terms of the infection risk. In another study with a similar study design, 308 RA patients were evaluated (denosumab and bDMARD combined: *n* = 102; bDMARD alone: *n* = 206). While three serious infections occurred in the group taking denosumab and bDMARD, four serious infections and one opportunistic infection occurred in the group taking bDMARD alone [24]. In a study conducted by Curtis et al. [10] of patients with RA, those receiving denosumab and bDMARD in combination were compared with patients receiving ZA and bDMARD in combination in terms of the risk of infection requiring hospitalisation. The crude rate of infections requiring hospitalisation per 100 person-years was 14.9 (95% CI 12.2–18.1) in the denosumab group and 13.9 (95% CI 12.5–15.4) in the ZA group. In conclusion, there was no significant increase in infections requiring hospitalisation in RA patients receiving denosumab compared with RA patients receiving ZA.

In our study, consistent with the literature, no significant increase in the risk of requiring hospitalisation or outpatient infections was observed in IRD patients receiving bDMARD or tsDMARD in combination with denosumab compared with IRD patients receiving bDMARD or tsDMARD in combination with ZA. However, the infection rates in the denosumab group (infection rate requiring hospitalisation: 13.6%; infection rate requiring outpatient treatment: 26.8%) were higher than stated in the literature. In a study by Lau et al. [24] involving 102 patients, three cases of serious infections were observed, while in a study by Mirzaei et al. [23], the infection rate in the denosumab group was 5%. In our study, there was a higher rate of comorbidities in the denosumab group than in the ZA group. The limited use of bisphosphonate-based OP treatments in patients with CKD [30] appears to be the main factor contributing to the high comorbidity rate in the denosumab group. Infection rates increase during the course of CKD and are a major cause of hospitalisation, especially in patients with end-stage renal failure [31]. Comorbidities such as CKD could be one of the reasons for the increased infection rates in our study. The fact that RTX is the most commonly used bDMARD treatment may also have contributed to this situation. In a study comparing RTX with anti-TNF agents, the incidence of serious infections was higher in the RTX group, although the rates of adverse events were similar [32]. This could also be the reason why the infection rates were higher in the ZA group. Again, the use of GCs increases the risk of infection even at low doses, and this risk may increase with the dosage [33,34]. In our study, the proportion of patients receiving low-dose GCs (≤5 mg prednisolone equivalent dose) was higher in the ZA group, and when analysing infection rates, it was higher than in the denosumab group. In addition, the use of a prednisolone equivalent dose of more than 5 mg in the denosumab group significantly increased the risk of infection. Again, the inclusion of other IRDs such as SLE, dermatomyositis and vasculitis and the possibility of more extra-articular involvement, such as renal involvement during the course of these diseases, may have contributed to the increased infection rates. However, our inclusion of patients with IRDs other than RA will contribute to the literature regarding the use of denosumab combinations despite the small number of patients in contrast to other studies.

### Limitations

The main limitations of our study are that it was conducted at a single centre, retrospectively and with a small number of patients. The short duration of the treatment also makes it difficult to draw conclusions about long-term risks. Furthermore, no comparison was made as to whether the patients were in remission or in the active phase and no sub-analysis was carried out regarding the risk of infection in these patients. Studies with a larger number of patients, a longer combined use duration and analyses of the bDMARD or tsDMARD agent subgroups used in this treatment are required.

## 5. Conclusions

In conclusion, there was no significant increase in infection rates in patients with IRD who received denosumab in combination with bDMARD or tsDMARD compared with patients with IRD who received ZA in combination with bDMARD or tsDMARD. For short-term use, the combination of denosumab with bDMARD or tsDMARD treatments may be safe.

## Figures and Tables

**Table 1:** Comparison of clinical and demographic characteristics of patients receiving denosumab and zoledronic acid.

	**Zoledronic Acid (ZA)**	**Denosumab**	***p***
	**n (%)** **30 (34.1)**	**n (%)** **58 (65.9)**	
**Age (years)**	59.07 (±SD: 13.65)	69.93 (±SD: 11.72)	***<0.001 ^m^***
**Gender**			
Female	22 (73.33)	50 (86.21)	0.138 ^χ2^
Male	8 (26.67)	8 (13.79)	
**RA**	14 (46.66)	42 (72.41)	***0.017*** ***^χ^*^2^**
**AS**	2 (6.66)	4 (6.89)	0.968 ^χ2^
**PSA**	2 (6.66)	2 (3.44)	0.603 ^χ2^
**SSC**	2 (6.66)	4 (6.89)	0.968 ^χ2^
**Vasculitis**	4 (13.33)	6 (10.34)	0.675 ^χ2^
**SLE**	2 (6.66)	0 (0.00)	0.114 ^χ2^
**DM**	4 (13.33)	0 (0.00)	***0.012 ^χ^*** **^2^**
**Duration of denosumab use (month)**		24 (6, 72)	
**Duration of ZA use (month)**	24 (12, 60)		
**Comorbidity**	14 (46.66)	44 (75.86)	***0.006 ^χ^*** **^2^**
Diabetes mellitus	4 (13.33)	10 (17.24)	0.635 ^χ2^
Hypertension	12 (40.00)	38 (65.51)	***0.022 ^χ^*** **^2^**
Coronary artery disease	0 (0.00)	8 (13.79)	***0.033 ^χ^*** **^2^**
Chronic kidney disease	0 (0.00)	12 (20.68)	***0.007 ^χ^*** **^2^**
Hyperlipidaemia	0 (0.00)	14 (24.13)	***0.003 ^χ^*** **^2^**
Congestive heart failure	0 (0.00)	4 (6.89)	0.295 ^χ2^
Interstitial lung disease	2 (6.66)	2 (3.44)	0.603 ^χ2^
**bDMARDs**			
Infliximab	2 (6.66)	2 (3.44)	0.603 ^χ2^
Duration of infliximab use (month)	0 (0, 30)	0 (0, 24)	0.464 ^m^
Abatacept	0 (0.00)	4 (6.89)	0.295 ^χ2^
Duration of abatacept (month)	0 (0, 0)	0 (0, 48)	0.143 ^m^
Adalimumab	2 (6.66)	6 (10.34)	0.569 ^χ2^
Duration of adalimumab (month)	0 (0, 12)	0 (0, 48)	0.548 ^m^
Certolizumab	2 (6.66)	2 (3.44)	0.603 ^χ2^
Duration of certolizumab (month)	0 (0, 12)	0 (0, 24)	0.526 ^m^
Etanercept	4 (13.33)	14 (24.13)	0.234 ^χ2^
Duration of etanercept (month)	0 (0, 120)	0 (0, 72)	0.271 ^m^
Golimumab	2 (6.66)	0 (0.00)	0.114 ^χ2^
Duration of golimumab (month)	0 (0, 12)	0 (0, 0)	***0.048 ^m^***
Rituximab	16 (53.33)	22 (37.93)	0.167 ^χ2^
Duration of rituximab (month)	0 (0, 84)	0 (0, 48)	***0.016 ^m^***
Tocilizumab	4 (13.33)	12 (20.68)	0.396 ^χ2^
Duration of tocilizumab (month)	0 (0, 36)	0 (0, 48)	0.432 ^m^
Secukinumab	2 (6.66)	2 (3.44)	0.603 ^χ2^
Duration of secukinumab (month)	0 (0, 3)	0 (0, 36)	0.526 ^m^
**tsDMARDs**			
Tofacitinib	4 (13.33)	6 (10.34)	0.675 ^χ2^
Duration of tofacitinib (month)	0 (0, 24)	0 (0, 72)	***0.041 ^m^***
**csDMARDs**			
Methotrexate	4 (13.33)	8 (13.79)	0.852 ^χ2^
Leflunomide	6 (20.00)	20 34.48)	0.158 ^χ2^
Sulfasalazine	4 (13.33)	6 (10.34)	0.675 ^χ2^
Hydroxychloroquine	14 (46.66)	18 (31.03)	0.148 ^χ2^
**Glucocorticoids**			
Prednisolone ≤ 5 mg equivalent dose	22 (73.33)	24 (41.37)	***0.004 ^χ^* ^2^**
Prednisolone > 5 mg equivalent dose	0 (0.00)	4 (6.89)	0.295 ^χ2^

AS, ankylosing spondylitis; bDMARDs, biologic disease-modifying antirheumatic drugs; csDMARDs, conventional synthetic disease-modifying antirheumatic drugs; DM, Dermatomyositis; PSA, psoriatic arthritis; RA, rheumatoid arthritis; SLE, systemic lupus erythematosus; SSC, systemic sclerosis; tsDMARDs, targeted synthetic disease-modifying antirheumatic drugs. Categorical data were expressed as n (%). Quantitative data were expressed as median (minimum, maximum) or mean (±SD). ^m^, Mann–Whitney u test; ^χ2^, Pearson chi-square test. *p* < 0.05: statistical significance level.

**Table 2:** Comparison of the use of bDMARDs, tsDMARDs and csDMARDs between denosumab and zoledronic acid groups after IPTW application.

	Zoledronic Acid (ZA)	Denosumab	*p*
	n (%)	n (%)	
	64 (44.5) *	81 (55.5) *	
**bDMARDs**			
Infliximab	2 (%3.1)	2 (%2.5)	1.000 ^f^
Abatacept	0 (%0)	6 (%7.4)	***0.034 ^f^***
Adalimumab	3 (%4.7)	7 (%8.6)	0.513 ^f^
Certolizumab	2 (%3.1)	7 (%8.5)	0.299 ^f^
Etanercept	5 (%7.8)	17 (%21.0)	***0.028 ^χ^* ^2^**
Golimumab	2 (%3.1)	0 (%0)	0.193 ^f^
Rituximab	31 (%48.4)	26 (%32.1)	***0.045 ^χ^* ^2^**
Tocilizumab	18 (%28.1)	21 (%25.6)	0.733 ^χ2^
Secukinumab	2 (%3.1)	2 (%2.5)	1.000 ^f^
**tsDMARDs**			
Tofacitinib	13 (%20.3)	6 (%7.4)	***0.022 ^χ^* ^2^**
**csDMARDs**			
Methotrexate	6 (%9.4)	11 (%13.6)	0.434 ^χ2^
Leflunomide	10 (%15.6)	34 (%42.0)	***0.001 ^χ^* ^2^**
Sulfasalazine	12 (%18.8)	9 (%11.0)	0.184 ^χ2^
Hydroxychloroquine	36 (%56.3)	28 (%34.6)	***0.009 ^χ^* ^2^**

bDMARDs, biologic disease-modifying antirheumatic drugs; csDMARDs, conventional synthetic disease-modifying antirheumatic drugs; tsDMARDs, targeted synthetic disease-modifying antirheumatic drugs. Categorical data were expressed as n (%). *p* < 0.05: statistical significance level. * Weighted sample size due to IPTW (Inverse Probability of Treatment Weighting); ^f^ Fisher and ^χ2^ Pearson chi-square test.

**Table 3:** Comparison of infections in patients receiving senosumab and zoledronic acid.

	Zoledronic Acid (ZA)	Denosumab	*p*
	n (%)	n (%)	
	65 (44.5) *	81 (55.5) *	
**Hospitalisation due to serious infection**	22 (65.6)	11 (13.6)	**0.003 ^χ2^**
Urinary tract infection	3 (4.6)	5 (6.2)	0.733 ^f^
Pneumonia	19 (29.7)	6 (7.4)	***<0.001 ^χ^* ^2^**
**Outpatient infection**	31 (48.4)	22 (26.8)	***0.007 ^χ^* ^2^**
Urinary tract infection	28 (43.8)	14 (17.3)	***<0.001 ^χ^* ^2^**
Pneumonia	3 (4.7)	2 (2.5)	0.655 ^f^
Cellulitis	0 (0.00)	2 (2.5)	0.503 ^f^
Gastroenteritis	0 (0.00)	3 (3.7)	0.255 ^f^

* Weighted sample size due to IPTW (Inverse Probability of Treatment Weighting). Categorical data were expressed as n (%). ^χ2^ Pearson and ^f^ Fisher chi-square test, *p* < 0.05: statistical significance level.

## Data Availability

The data presented in this study are available on request from the corresponding author.

## Abbreviations

biologic disease-modifying antirheumatic drugs (bDMARDs), coronary artery disease (CAD), Confidence Interval (CI), conventional disease-modifying antirheumatic drugs (csDMARDs), chronic kidney disease (CKD), chronic renal disease (CRD), dermatomyositis (DM), glucocorticoids (GCs), Hazard Ratio (HR), hypertension (HT), immune-mediated inflammatory disease (IMID), inflammatory rheumatic disease (IRD), inverse probability of treatment weighting (IPTW), osteoporosis (OP), osteoprotegerin (OPG), receptor activator of nuclear factor-κB (RANK), receptor activator of nuclear factor-κB ligand (RANKL), rheumatoid arthritis (RA), rituximab (RTX), systemic lupus erythematosus (SLE), systemic sclerosis (SSC), targeted synthetic disease-modifying antirheumatic drugs (tsDMARDs), tumour necrosis factor (TNF) and zoledronic acid (ZA).
